# Partial purification and functional characterization of Ts19 Frag-I, a novel toxin from *Tityus serrulatus* scorpion venom

**DOI:** 10.1186/s40409-015-0051-6

**Published:** 2015-12-01

**Authors:** Priscila C. Lima, Karla C. F. Bordon, Manuela B. Pucca, Felipe A. Cerni, Karina F. Zoccal, Lucia H. Faccioli, Eliane C. Arantes

**Affiliations:** Department of Physics and Chemistry, School of Pharmaceutical Sciences of Ribeirão Preto, University of São Paulo (USP), Ribeirão Preto, SP Brazil; Department of Clinical Analyses, Toxicology and Food Sciences, School of Pharmaceutical Sciences of Ribeirão Preto, University of São Paulo (USP), Ribeirão Preto, SP Brazil; Departamento de Física e Química, Faculdade de Ciências Farmacêuticas de Ribeirão Preto, Universidade de São Paulo (USP), Avenida do Café, s/n, Ribeirão Preto, SP 14.040-903 Brazil

**Keywords:** β-KTx, cytokine, interleukin, neurotoxin, pro-inflammatory, scorpion venom, *Tityus serrulatus*

## Abstract

**Background:**

The yellow scorpion *Tityus serrulatus* (Ts) is responsible for the highest number of accidents and the most severe scorpion envenoming in Brazil. Although its venom has been studied since the 1950s, it presents a number of orphan peptides that have not been studied so far. The objective of our research was to isolate and identify the components present in the fractions VIIIA and VIIIB of Ts venom, in order to search for a novel toxin. The major isolated toxins were further investigated for macrophage modulation.

**Methods:**

The fractions VIIIA and VIIIB, obtained from Ts venom cation exchange chromatography, were rechromatographed on a C18 column (4.6 × 250 mm) followed by a reversed-phase chromatography using another C18 column (2.1 × 250 mm). The main eluted peaks were analyzed by MALDI-TOF and Edman’s degradation and tested on macrophages.

**Results:**

The previously described toxins Ts2, Ts3-KS, Ts4, Ts8, Ts8 propeptide, Ts19 Frag-II and the novel peptide Ts19 Frag-I were isolated from the fractions VIIIA and VIIIB. Ts19 Frag-I, presenting 58 amino acid residues, a mass of 6,575 Da and a theoretical pI of 8.57, shares high sequence identity with potassium channel toxins (KTx). The toxins Ts4, Ts3-KS and the partially purified Ts19 Frag-I did not produce cytotoxic effects on macrophage murine cells line (J774.1). On the other hand, Ts19 Frag-I induced the release of nitric oxide (NO) by macrophages, while Ts4 and Ts3-KS did not affect the NO production at the tested concentration (50 μg/mL). At the same concentration, Ts19 Frag-I and Ts3-KS increased the production of interleukin-6 (IL-6). Ts19 Frag-I and Ts4 did not induce the release of IL-10, IL-1β or tumor necrosis factor-α by macrophage cells using the tested concentration (50 μg/mL).

**Conclusions:**

We partially purified and determined the complete sequence and chemical/physical parameters of a new β-KTx, denominated Ts19 Frag-I. The toxins Ts4, Ts3-KS and Ts19 Frag-I showed no cytotoxicity toward macrophages and induced IL-6 release. Ts19 Frag-I also induced the release of NO, suggesting a pro-inflammatory activity.

## Background

*Tityus serrulatus* venom (Tsv) is composed of insoluble mucus, neurotoxic proteins that affect sodium or potassium channels, bioactive amines, hypotensins, proteinases, hyaluronidases, a bradykinin-potentiating peptide, a kallikrein inhibitor, allergenic proteins and other peptides whose biological functions are still not known [1]. It is estimated that Tsv contains over 300 different toxins [2].

Neurotoxins are the most studied components of Tsv because of their interactions with ionic channels in excitable membranes and their role in the envenoming [3]. Tsv neurotoxins are represented by long-chain Na^+^-channel toxins (NaTx) and short-chain K^+^-channel toxins (KTx) [1].

The family of potassium channels is comprised of the largest number of ion channels subtypes with high structural and functional diversities [4]. These channels are involved in several pathologies, e.g., asthma, cardiac arrhythmia, T-cell-mediated autoimmune disease, immune response to infection and inflammation, and hypertension [5].

KTx are classified into four families: α, toxins constituted by 23-43 amino acids linked by 3-4 disulfide bonds; β, long peptides (~60 amino acid residues) stabilized by three disulfide bonds; γ, ether-a-go-go (ERG) channel blockers with 36-47 amino acid residues connected by 3 or 4 disulfide bonds; and κ, poor K^+^ blockers with two α-helices stabilized by two disulfide bonds [6]. Moreover, some KTx, whose N-terminal region starts with KIK residues, may show cytolytic, antimicrobial and hemolytic activities [7, 8]. Among the Tsv toxins, Ts6, Ts7, Ts9, Ts15 and Ts16 are classified as α-KTxs, while Ts8 and Ts19 are classified as β-KTxs [1].

Scorpion venoms and their isolated toxins are responsible for several immunological properties (e.g., inflammation) observed after scorpion envenoming [9–11]. Neurotoxins specific for voltage-gated K^+^ and Na^+^ channels can affect many cells, such as macrophages, which participate in the inflammatory response of Ts envenoming [12, 13]. Intense activation of the immune system by pro-inflammatory cytokines, such as IL-6 and tumor necrosis factor-α (TNF-α), is observed after the Ts envenoming [14]. Furthermore, molecules from venoms that can be recognized by the pattern recognition receptors (PRRs) of macrophages were recently denominated the venom-associated molecular pattern (VAMP) [15]. Tsv also induces the formation of lipid bodies (LBs) and generates PGE_2_ and LTB_4_ through TLR2 and TLR4 stimulation and peroxisome proliferator-activated receptor gamma (PPAR-γ) activation [16].

Until now, only the effects of few Ts toxins – namely of Ts1, Ts2, Ts5 and Ts6 – have been evaluated for macrophage activation [17–19].

Therefore, the present work purified the components present in the fractions VIIIA and VIIIB from *Tityus serrulatus* venom. The major eluted peaks were analyzed by MALDI-TOF mass spectrometry and had their N-terminal sequence determined by Edman degradation. Additionally, the effect of a new β-KTx – Ts19 Frag-I, Ts4 and Ts3-KS were investigated for their cytotoxicity and cytokines and NO production on macrophages.

## Methods

### Isolation of toxins present in the fractions VIIIA and VIIIB from Tsv

Tsv was provided by the vivarium at the School of Medicine of Ribeirão Preto, University of São Paulo, Brazil, after extraction by the electrical stimulation method using 12 mV [20]. Desiccated Tsv (50 mg) was purified through cation exchange chromatography using an FPLC system, as described by Cerni *et al*. [21]. The fractions VIIIA and VIIIB (4 mg) were submitted to reversed-phase chromatography using a 4.6 mm × 250.0 mm C18 column (5 μm particles, Shimadzu Corp., Japan); the eluted subfractions were rechromatographed on a 2.1 mm × 250.0 mm C18 column (3.6 μm particles, Phenomenex, USA). Both reversed-phase columns were equilibrated with 0.1 % (V/V) trifluoroacetic acid (TFA) and the subfractions were eluted using a concentration gradient from 0 to 100 % of solution B (80 % acetonitrile in 0.1% TFA). Absorbance was automatically registered at 214 nm by the FPLC Äkta Purifier UPC-10 system (GE Healthcare, Sweden).

### N-terminal sequencing

The amino acid residues of the N-terminal region from the eluted subfractions were sequenced by Edman degradation [22] on an automated sequencer model PPSQ-33A (Shimadzu Co., Japan). The identities of the sequenced peptides were analyzed using BLAST [23]. The complete primary sequences were retrieved from the Universal Protein Resource Knowledgebase [24]. The ProtParam tool [25] was used to estimate the pI of new toxins. The predicted molecular masses were determined using the Sequence Editor 3.2 program.

### MALDI-TOF mass spectrometry

The eluted subfractions were submitted to matrix-assisted laser desorption/ionization (MALDI) time-of-flight (TOF) mass spectrometry (UltrafleXtreme, Bruker Daltonics, USA). The mass spectra were obtained in the linear positive mode after the equipment was calibrated with a standard peptide (Bruker peptide calibration standard II). Samples were resuspended in a solution of 80 % (V/V) acetonitrile (ACN) and 0.2 % (V/V) TFA and combined at the ratio 1:1 with 5 mg/mL 2,5-dihydroxybenzoic acid (DHB) matrix.

### Murine macrophage cell line J774.1 culture

The macrophage cell line J774.1 was obtained from the American Type Culture Collection (ATCC, USA). The cells were grown, total number of cells was counted, viability was determined and cells were plated, as previously described [17].

### Cytotoxicity assay

The toxins (50 μg/mL) isolated from fractions VIIIA and VIIIB were incubated with the J774.1 macrophage line cells for 24 h. Then, the cell viability was evaluated using the 3-(4,5-dimethylthiazol-2-yl)-2,5-diphenyltetrazolium bromide (MTT) colorimetric assay (Sigma-Aldrich) [26], as described by Zoccal *et al*. [17]. The assay was performed in quadruplicate and the cytotoxicity of the toxins was measured at 570 nm. The results were expressed as a relative percentage of the cytotoxicity observed in the unstimulated control cells. The same concentration (50 μg/mL) was used in all the following assays. This concentration was chosen because a statistically significant effect on macrophage immunomodulation was previously observed using other Ts toxins at the same concentrations [17].

### Nitric oxide (NO) release

The amount of nitrite (NO^2−^) in the supernatants was measured, at 540 nm, as an indicator of NO production according to the Griess method [27]. The assay was performed in quadruplicate from two independent experiments.

### Release of cytokines

The concentrations of the cytokines TNF-α, IL-1β, IL-6 and IL-10 in culture supernatants were quantified by enzyme-linked immunoabsorbent assay (ELISA) using specific antibodies and quantified at 405 nm, as previously described [17]. The sensitivities were > 10 pg/mL. The assays were performed in quadruplicate.

### Statistical analysis

Data are expressed as mean ± standard error of mean (SEM) and were analyzed using one-way ANOVA. Values of *p* < 0.05 were considered statistically significant.

## Results

### Isolation of toxins present in the fractions VIIIA and VIIIB from Tsv

**Fig. 1:**
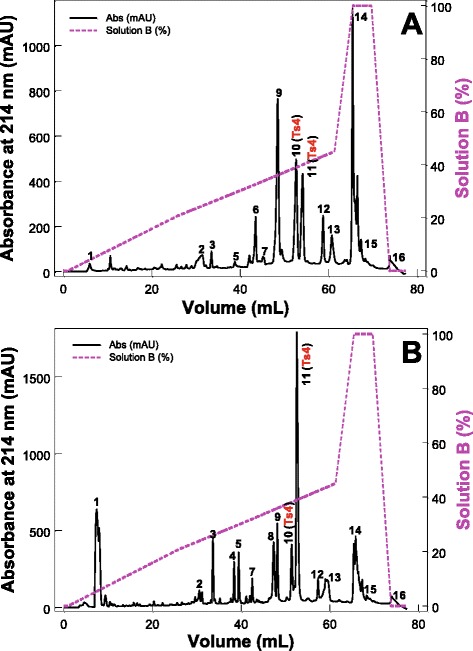
Chromatographic profiles of fractions VIIIA and VIIIB from Tsv. (**a**) Fraction VIIIA. (**b**) Fraction VIIIB. Fractions (4 mg, eluted of the cation exchange chromatography from *Tityus serrulatus* venom) were submitted to RP-FPLC on a C18 column (4.6 mm × 250.0 mm, 5 μm particles, Shimadzu Corp.). The column was equilibrated with 0.1 % trifluoroacetic acid (TFA) and the proteins were eluted using a concentration gradient from 0 to 100 % of solution B (80 % acetonitrile (ACN) in 0.1 % TFA), represented by the dashed line. Absorbance was monitored at 214 nm, at 25 °C, using an FPLC Äkta Purifier UPC-10 system. Fractions of 0.3 mL/tube were collected at a flow rate of 0.7 mL/min

The fractions VIIIA and VIIIB, eluted from the cation exchange chromatography of Tsv, present low resolution in this chromatographic step (data not shown). For this reason, to isolate the toxins, these fractions were submitted to reversed-phase fast protein liquid chromatography (RP-FPLC) on a C18 column (Fig. 1 – a and b). The subfractions eluted from fraction VIIIA that presented the same retention time from those eluted from the fraction VIIIB were designed with the same number. The subfractions 4 and 8 did not elute from the fraction VIIIA (Fig. 1 – a), while a greater number of subfractions eluted from the fraction VIIIB under the same chromatographic conditions, ranging from 1 to 16 (Fig. 1 – b).

**Fig. 2:**
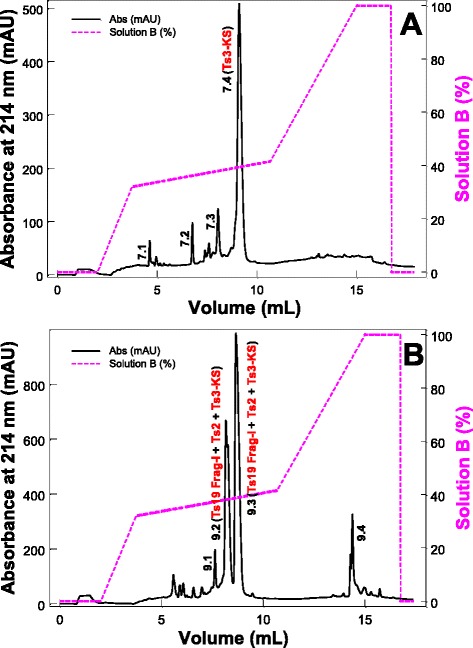
Rechromatography of the subfractions eluted from the fractions VIIIA and VIIIB. (**a**) Subfraction 7. (**b**) Subfraction 9. The C18 column (2.1 mm × 250.0 mm, 3.6 μm particles, Phenomenex) was equilibrated with 0.1 % TFA and the proteins were eluted using a concentration gradient from 0 to 100 % of solution B (80 % ACN in 0.1 % TFA), represented by the dashed line. Absorbance was monitored at 214 nm, at 25 °C, using a FPLC Äkta Purifier UPC-10 system. Fractions of 0.3 mL/tube were collected at a flow rate of 0.4 mL/min

The subfractions 7 and 9 were rechromatographed on a C18 column (2.1 × 250 mm, 3.6 μm particles) (Fig. 2 – a and b) and their components were used in the next assays.

### N-terminal sequencing and *in silico* analysis

The primary sequences of the subfractions 6-13 and peaks 9.2 and 9.3 were determined by Edman degradation resulting in the identification of the peptides Ts2, Ts3, Ts4, Ts8, Ts8 propeptide, Ts19 Frag-I and Ts19 Frag-II present in the fractions VIIIA and VIIIB (Table **1**).

**Table 1:** N-terminal sequence of the main peaks eluted from the chromatographic steps. Assignment of the peaks to protein families by BLAST against a *Tityus* venom database

Peak	N-terminal sequence	Protein family (Uniprot ID)
6	KDKMKAGWERLTSQSEYACP…	Ts19 Frag-II (P86822)
	KIKEKIIEAKDKMKAGWERL…	Ts19 Frag-I (P86822)
7	KKDGYPVEYDNCAYICWNYDNAY…	Ts3 (P01496)
	KDKMKAGWERLTSQSEYACPAID…	Ts19 Frag-II (P86822)
8	KIKEKIIEAKDKMKAGWERLTSQSEYACPAIDKFCEDHCAAKKAVGKCDDFKCNCIK	Ts19 Frag-I (P86822)
	KEGYAMDHEGCKFSCFIRPAGFCDGYCKTHLKASSGYCAWPACYCYGVPD…	Ts2 (P68410)
9	KIKEKIIEAKDKMKAGWERLTSQSEYA…	Ts19 Frag-I (P86822)
	KKDGYPVEYDNCAYICWNYDNAYCDKL…	Ts3 (P01496)
	KEGYAMDHEGCKFSCFIRPAGFCDGYC…	Ts2 (P68410)
10	GREGYPADSKGCKITCFLTAAGYCNTECTL…	Ts4 (P45669)
11	GREGYPADSKGCKITCFLTA…	Ts4 (P45669)
12	GREGY…	Ts4 (P45669)
13	KLVALIPNDQLRSILKAVVHKVAKTQFGCPAYEGYCNDHCNDIERKDG…	Ts8 (P69940)
	GLREKHVQKLVALIPNDQLRSILKAVVHKVAKTQFGCPAYEGYCNDHC…	Ts8 propeptide (P69940)
	GREGYPADSKG…	Ts4 (P45669)
9.2	KIKEKIIEAK…	Ts19 Frag-I (P86822)
	KEGYAMDHEG…	Ts2 (P68410)
9.3	KIKEKIIEAKDKMKA…	Ts19 Frag-I (P86822)
	KEGYAMDHEGCKFSC…	Ts2 (P68410)

…, the primary sequence was not completely determined

Ts19 Frag-I, identified in the peaks 6, 8 and 9, and partially purified in the peaks 9.2 and 9.3, was recently deposited in the UniProt data bank by our group [28]. It was possible to sequence 57 amino acid residues of this toxin by Edman degradation, including six cysteine residues. This primary sequence was analyzed by the program Sequence Editor 3.2 and the molecular mass of the oxidized monoisotopic toxin (S-S) was calculated as 6,458 Da.

### MALDI-TOF mass spectrometry

**Fig. 3:**
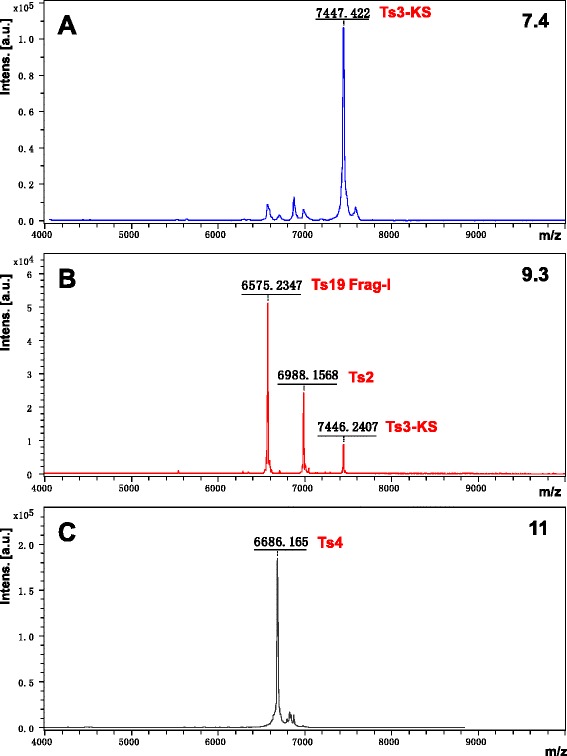
Mass spectra of the peaks (**a**) 7.4, (**b**) 9.3 and (**c**) 11. The mass spectra were obtained by MALDI-TOF mass spectrometry in a positive linear mode using DHB matrix

The peaks 7.4, 9.3 and subfraction 11 had their molecular masses determined through mass spectrometry (Fig. 3 – a to c). The mass spectra of the peak 7.4 and subfraction 11 showed respective main peaks of 7,447.4 Da and 6,683.2 Da (Fig. 3 – a and c). The peak 9.3 was mainly represented by Ts19 Frag-I (63.7 %) with a mass of 6,570.0 Da (Fig. 3 – b). It presented contaminants of 6,985.2 Da and 7,441.5 Da (Fig. 3 – b), which correspond to 25.7 % and 10.6 % of the peak 9.3, respectively.

### Effect of the toxins on macrophage viability

**Fig. 4:**
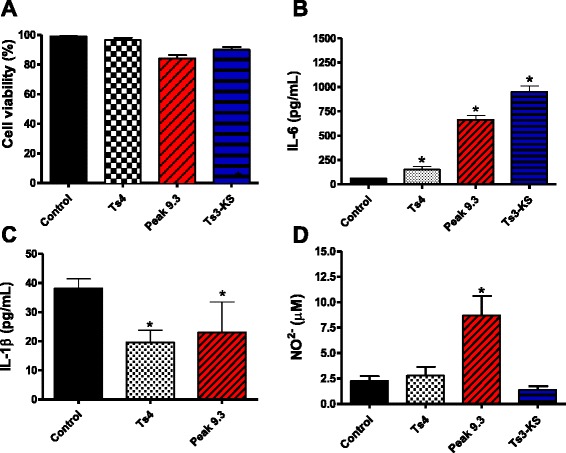
Effects of Ts4, Ts3-KS and peak 9.3^#^ on macrophage viability and the cytokine and NO production. Adherent cells were stimulated with Ts4, Ts3-KS and peak 9.3 (50 μg/mL) for 24 h in 5 % CO_2_ at 37 °C. The supernatants were collected after 24 h. (**a**) Cell viability was measured by MTT assay. Each column represents the mean ± SEM (*n* = 6), and the data are from two independent set of experiments (*p < 0.05 compared to control, non-stimulated cells). The concentrations of the cytokines (**b**) IL-6 and (**c**) IL-1β in the supernatants were determined by ELISA. The amount of (**d**) NO^2−^ present in the supernatant was determined by the Griess method. Values are expressed as mean ± SD (*n* = 4). *p < 0.05 compared to control, non-stimulated cells (ANOVA and Dunnett's post-test). ^#^Peak 9.3: Ts19 Frag-I contaminated with Ts2 and Ts3-KS

The toxicity of the toxins Ts3-KS (peak 7.4), Ts19 Frag-I (peak 9.3) and Ts4 (peak 11) at 50 μg/mL was analyzed by MTT assay. We demonstrated that these toxins did not affect J774.1 cell viability when compared to non-stimulated cells (Fig. 4 – a).

### Effects of the toxins on NO and cytokines production

The toxins Ts4 and Ts3-KS (50 μg/mL) did not induce NO production when compared to non-stimulated cells (control). However, cells stimulated with peak 9.3 (50 μg/mL; Ts19 Frag-I contaminated with Ts2 and Ts3-KS) induced NO production by J774.1 cells (*p* < 0.05) (Fig. 4 – b).

The ability of the toxins (Ts4 and peak 9.3) to stimulate macrophages was investigated through the production of cytokines. Ts3-KS was only tested for IL-6 production because of the low sample quantity. Ts4, Ts3-KS and peak 9.3 at 50 μg/mL induced IL-6 production (*p* < 0.05) (Fig. 4 – c), while the toxins Ts4 and Ts19 Frag-I did not show a significant effect compared to control on IL-10 and TNF-α (data not shown). Ts4 and peak 9.3 also significantly inhibited the IL-1β production (Fig. 4 – d).

## Discussion

The components obtained from fractions VIIIA and VIIIB were analyzed through MALDI-TOF mass spectrometry and Edman degradation. Among the identified toxins are Ts2, Ts3-KS, Ts4, Ts8, Ts8 propeptide, Ts19 Frag-II and a novel partially purified β-KTx, denominated Ts19 Frag-I.

Ts2 (also known as TsTX-III, TsTX-II; *Tityus* toxin II or toxin T1-IV) presents features of β-NaTx but with α-like activity [29]. Ts2 stimulated the production of IL-10, suggesting the presentation of an anti-inflammatory activity by this toxin [17].

The precursor of the α-NaTx Ts3 (previously known as TsTX, Tityustoxin or TsIV-5), containing the sequence Gly-Lys-Lys in the C-terminal region, is processed by carboxypeptidases that remove the Lys residues. The remaining Gly-extended peptide is converted into a des-Gly peptide amine by an α-amidating enzyme to produce a serine-amide in its C-terminal end [30], herein denominated Ts3-KS. However, the biological role of this post-translational modification remains unclear [1].

Ts8 (also known as Tityustoxin K-beta or TsTx-kappa beta) was the first described member of β-KTx subfamily and was characterized as a selective blocker of voltage-gated non-inactivation K^+^ channels in synaptosome preparations [31]. Its mature chain is comprised of 60 amino acid residues, while the Ts8 propeptide contains an additional eight amino acid residues in its N-terminal region [7].

Additionally, Ts4 (also known as TsTX-VI, Tityustoxin-6, Tityustoxin VI, TsTXVI, toxin VI, Ts VI and TsNTxP), was the main toxin eluted from the fraction VIIIB, although it is also present in a high proportion in the fraction VIIIA. Ts4 causes allergic reaction, lachrymation, spasm of the hind legs in mice and dose dependent neurotransmitter release [3].

The α-KTx Ts6 induced NO and IL-6 production and inhibited the release of TNF-α [17]. Kaliotoxin 2 (KTX_2_), an α-KTx from *Androctonus australis hector* scorpion venom, induces severe alterations in hepatic and pancreatic tissues by the activation of the inflammatory response with release of IL-6 and TNF-α [32]. However, there is no previously published study on the effect of β-KTx on macrophages. In the present work, a novel β-KTx, named Ts19 Frag-I, was partially isolated and its effects on macrophage immunomodulation were evaluated.

In 2008, 27 amino acids residues of a new β-Ktx-like toxin from Tsv were identified by peptidomic analysis, whose precursor, known as Ts19, was determined through a transcriptomic study of the Ts venom gland [33, 34]. Posteriorly, two mature fragments of Ts19, named Ts19 Frag-I and Ts19 Frag-II, were deposited in the UniProt databank [28; Swiss-Prot: P86822]. The post-translational engineering of Ts19 toxin and its fragments, named post-splitting, has been recently suggested. Moreover, Ts19 Frag-II presents a specific and significant blocking effect on Kv1.2 [35].

**Fig. 5:**
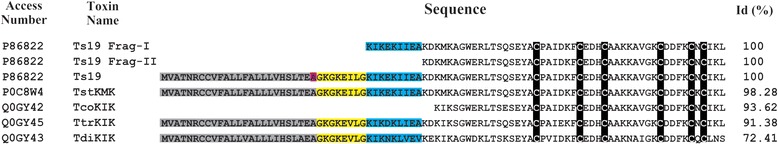
Ts19 Frag-I alignment. The multiple sequence alignment of Ts19 Frag-I with other β-KTx class (subfamily) 2 scorpion toxins: the amino acid sequences are highlighted according to the residues responsible for signal peptide (gray), propeptide (yellow) and cytolytic effect (blue). The amino acid in pink is considered the N-terminal residue of the toxin by Alvarenga *et al*. [34]. The alignments and identity – Id (%) were performed using ClustalW2. Cysteines are highlighted in black

The corresponding molecular mass of the 57 amino acid residues of oxidized monoisotopic toxin (S-S) Ts19 Frag-I (peak 9.3) sequenced through Edman degradation was calculated as 6,458 Da. The average molecular mass of the same peak was determined as 6,575 Da through MALDI-TOF mass spectrometry, linear mode. The difference between these masses corresponds to the amino acid residue (Leu or Ile) of the C-terminal region. Since the Ts19 Frag-I shares high identity with the β-KTx-like toxins TstKMK from *T. stigmurus* and TtrKIK from *T. trivittatus* and with Ts19, which presents a Leu in the C-terminal, we deduced that the amino acid residue to complete the entire sequence from Ts19 Frag-I is Leu. These 58 amino acid residues were submitted to ProtParam, a tool that predicted the pI 8.57. The composition of Ts19 Frag-I contains a high content of Lys residues, which explains the predicted basic isoeletric point. A similar result was observed experimentally with Ts15 [36]. The theoretical mass of oxidized monoisotopic (S-S) Ts19 Frag-I (peak 9.3) calculated by the Sequence Editor was 6,571 Da, indicating the six cysteine residues that form three disulfide bonds, as observed in the β-KTx family [6]. Ts19 Frag-I was classified into the β-KTx class (subfamily) 2, since it shares high similarity with other β-KTxs belonging to this class (Fig. 5).

The Ts19 Frag-I presents nine additional amino acid residues in the N-terminal region when compared with Ts19 Frag-II. Interestingly, the N-terminal region of Ts19 Frag-I starts with the amino acid residues KIK. Other toxins that have KIK in their N-terminal region showed cytolytic, antimicrobial and hemolytic activities [7, 8]. The Ts19 Frag-II identified in the fractions VIIIA and VIIIB from Ts (the present work) was previously identified in the fractionation of Tsv on a C18 column and corresponds to 0.8 to 1.8 % of the total venom protein [37].

The peak 9.3 is constituted mainly (63.7 %) by Ts19 Frag-I (6,570.0 Da) and by peptides of 6,985.2 Da and 7,441.5 Da, whose N-terminal sequences corresponded to Ts2 and Ts3-KS, respectively. The respective theoretical molecular masses of oxidized monoisotopic (S-S) Ts2 and Ts3-KS calculated by the Sequence Editor are 6,985 Da and 7,442 Da [1], confirming that the proteins identified by Edman degradation are correct.

The N-terminal of the peak 7.4 identified the toxin Ts3-KS. Its oxidized monoisotopic (S-S) molecular mass corresponds to 7,442 Da [1] while the mass spectrum showed 7,447.4 Da, confirming that the peak 7.4 is Ts3-KS. The N-terminal of the subfraction 11 permitted identification of the toxin Ts4, whose oxidized monoisotopic (S-S) molecular mass of 6,704 Da [1]. The molecular mass of 6,683.2 Da determined through mass spectrometry confirmed that the subfraction 11 is Ts4.

The toxins Ts3-KS (peak 7.4), peak 9.3 (Ts19 Frag-I) and Ts4 (peak 11) did not affect macrophage viability. In relation to cytokine modulation in macrophages, all tested toxins stimulated IL-6 production, although Ts3-KS proved to be the most potent stimulus. However, Ts3-KS and peak 9.3 did not change TNF-α production. Based on the peak 9.3 components (Ts2, Ts3-KS and Ts19 Frag-I), we eliminate the Ts2 participation in the peak stimulus since Ts2 is a potent inductor of TNF-α release even with low concentration (25 μg/mL) [17]. Moreover, corroborating this statement, macrophages stimulated with Ts2 (25-100 μg/mL) did not induce the release of IL-6 [17]. As to Ts3-KS, this cytokine was able to increase IL-6 release by macrophages and may have contributed to the effect produced by the peak 9.3, even though Ts19 Frag-I is indicated as the major component of the peak by mass spectrometry and sequence analysis. Interestingly, Ts4 and peak 9.3 inhibited macrophage IL-1β production.

The cytokines IL-6, IL-1, and TNF-α are elevated in most inflammatory states and have been recognized as targets of therapeutic intervention [38]. On the other hand, IL-6 has already been implicated in anti-inflammatory responses [39]. Although only few cell types express the IL-6 receptor and respond to IL-6 cytokine, all cells can be stimulated via a soluble IL-6 receptor. Apparently, IL-6 performs regenerative and anti-inflammatory functions whereas the IL-6 receptor is pro-inflammatory [39]. Therefore, IL-6 can no longer be uniquely related to pro-inflammatory response.

In relation to IL-1β, the significant inhibition of this cytokine by Ts4 and peak 9.3 is highly interesting. In fact, Ts4 was considered non-toxic to mice due to its inability to induce the characteristics symptoms of toxicity produced by other scorpion toxins [40]. However, Ts4 can induce an allergic reaction and produce a dose-dependent neurotransmitter release (GABA and Glu) from synaptosomes [41]. Therefore, the inhibition of IL-1β and the lowest release of IL-6 compared with other toxins could explain the absence of symptomatology produced by Ts4. Likewise, peak 9.3 was also a potent inhibitor of IL-1β. Considering that Ts19 Frag-I is the main component of the peak and that this toxin is a β-KTx toxin (normally Kv blockers), a toxin class heretofore untested on macrophage modulation, a different effect is expected compared to classical Nav channel pro-inflammatory toxins (e.g., Ts1).

Finally, the NO release induced by peak 9.3 was highly groundbreaking. Ts6 toxin was the only known Ts toxin capable of stimulating this mediator release [17]. Although Ts6 and Ts19 Frag-I are toxins that act on K^+^ channels, they belong to different classes: α-KTx and β-KTx to Ts6 and Ts19 Frag-I, respectively [21]. Based on the results of isolated Ts3-KS (non-effect on NO modulation) and the fact that Ts2 (25-100 μg/mL) inhibited the release of NO, we conclude herein that Ts19 Frag-I is responsible for peak 9.3 macrophage modulation [17].

Based on the literature, high NO levels in the serum or in peritoneal macrophage culture supernatants may be associated with such severe conditions as septic shock, hypertension and severe envenoming [17, 42]. Thus, the effect of β-KTx toxins on pro-inflammatory response via NO and IL-6 should be further studied by our group to understand the participation of this toxin class on scorpion envenoming. Furthermore, Ts19 Frag-I could be used as a pharmacological tool to study cell NO signaling.

## Conclusions

The toxins Ts2, Ts3-KS, Ts4, Ts8, Ts8 propeptide and Ts19 Frag-II, and a novel partially purified putative β-KTx, denominated Ts19 Frag-I, were isolated from the fractions VIIIA and VIIIB from Ts venom and analyzed through MALDI-TOF mass spectrometry and Edman degradation. The toxins Ts4, Ts3-KS and Ts19 Frag-I induce the release of IL-6 and do not show cytolytic activity. Additionally, Ts19 Frag-I induces the release of NO in macrophage cells. These results may contribute to elucidating not only the knowledge of macrophage immunomodulation after scorpion envenoming but also to the inflammatory actions of Ts toxins.

## Abbreviations

ACN: acetonitrile
ATCC: American Type Culture Collection
BLAST: Basic Local Alignment Search Tool
DHB: dihydroxybenzoic acid
ELISA: enzyme-linked immunoabsorbent assay
ERG: ether-a-go-go channel
FPLC: fast protein liquid chromatography
frag.: fragment
IL: interleukin
KTx: K^+^-channel toxins
LBs: lipid bodies
MALDI-TOF: matrix-assisted laser desorption ionization time of flight
MTT: 3-(4,5-dimethylthiazol-2-yl)-2,5-diphenyltetrazolium bromide
NaTx: Na^+^-channel toxins
Nav: voltage-gated sodium channel
NO: nitric oxide (NO)
PPAR-γ: peroxisome proliferator-activated receptor gamma
PRRs: pattern recognition receptors
SEM: standard error of mean
TFA: trifluoroacetic acid
TNF: tumor necrosis factor
Ts: *Tityus serrulatus*
Tsv: *Tityus serrulatus* venom
VAMP: venom-associated molecular pattern
